# Identifying Transgenic Approaches to Mitigate Vector Transmission of Malaria by Anopheles gambiae

**DOI:** 10.7759/cureus.113451

**Published:** 2026-07-27

**Authors:** Scott T Kilian, Samuel A Cerezo, Rebecca L Sanchez

**Affiliations:** 1 Department of Applied Biomedical Sciences, University of the Incarnate Word School of Osteopathic Medicine, San Antonio, USA; 2 Department of Entomology, Texas A&M University, College Station, USA

**Keywords:** anopheles, gene drive, malaria, transgenic, vector transmission

## Abstract

Malaria, historically and contemporarily, poses a global health threat. As humans have engaged in control efforts with mosquitoes, the selection pressures placed upon mosquitoes have furthered the development of resistance to typical pyrethroids and other insecticides. Common management strategies such as insecticide-treated nets and residual spraying are proving to be more unreliable. As a result, novel control strategies must be developed to address the arms race between humans and the primary malaria vector, *Anopheles gambiae*. The introduction of transgenic mosquitoes and the use of Wolbachia offer techniques that decrease the use of chemical means of control. These techniques offer specifically targeted approaches to managing vector populations while avoiding further insecticide resistance. In this review, we delve into novel control strategies currently being used for *A. gambiae* and consider potential barriers for their use. A focused literature search was conducted using PubMed and Google Scholar with combinations of the terms “Anopheles gambiae”, “malaria vector”, “gene drive”, “Wolbachia”, “CRISPR”, and “transgenic mosquitoes”, with emphasis on studies published in the last 10 years.

## Introduction and background

A focused narrative literature review was conducted using PubMed and Google Scholar to identify peer-reviewed publications examining genetic approaches to malaria vector control in *Anopheles gambiae*. Searches were performed using combinations of the keywords "Anopheles gambiae", "malaria vector", "gene drive", "CRISPR", "Wolbachia", and "transgenic mosquitoes", with Boolean operators used to refine results. Articles published primarily between 2015 and 2025 were prioritized to capture recent advances, although seminal studies published before this period were included when they provided foundational information. 

Titles and abstracts were screened for relevance, followed by full-text review of potentially eligible articles. Eligible studies included original research articles and high-quality review articles addressing genetic vector control strategies, gene-editing technologies, Wolbachia-based approaches, ecological impacts, and implementation challenges related to malaria prevention. Editorials, opinion articles, conference abstracts, and studies not directly related to *A. gambiae* or malaria vector control were excluded. 

Relevant information extracted from each study included intervention type, study design, key findings, reported advantages and limitations, and implications for malaria control. Findings were synthesized qualitatively into thematic categories rather than quantitatively pooled. Because this work was conducted as a focused narrative review rather than a systematic review or meta-analysis, a formal risk-of-bias assessment was not performed. Figures illustrating mosquito biology and gene-editing concepts were created using BioRender. 

Malaria is a life-threatening disease that poses a risk to nearly half of the world’s population. The disease manifests through the infection of five different Plasmodium parasite species. The greatest risk to humans is posed by *Plasmodium falciparum* and *P. vivax* [1]. Plasmodium is vectored by female Anopheles mosquitoes, with *A. gambiae* being the most significant vector for those most at risk of malaria. Developing cost-effective approaches to mitigate and possibly eliminate malarial transmission is at the forefront of public health concerns in areas where transmission cycles of malaria are endemic. Measures of using interventions must be sensitive to cultural values and regulatory guidelines while maximizing the usage of funding to elicit the greatest pragmatic improvements to public health. This paper will attempt to identify the most cost-effective approaches to lower malaria infections by *A. gambiae* mosquitoes on a large scale by reviewing techniques currently under investigation and/or being used in endemic areas. 

Distributed in Sub-Saharan Africa, *A. gambiae* breeds in small pools of water that form due to rainfall [2]. Typically considered endophagic and endophilic [3], *A. gambiae* anthropophilic behaviors have been demonstrated as more likely to take place indoors rather than outdoors, with this behavior occurring more frequently during the dry season than the rainy season [4]. *A. gambiae* have also been demonstrated to have crepuscular feeding tendencies with peak biting hours being recorded between 18:00-22:00 hours and 04:00-06:00 hours, which is roughly sunset and sunrise hours [5].

**Figure 1:**
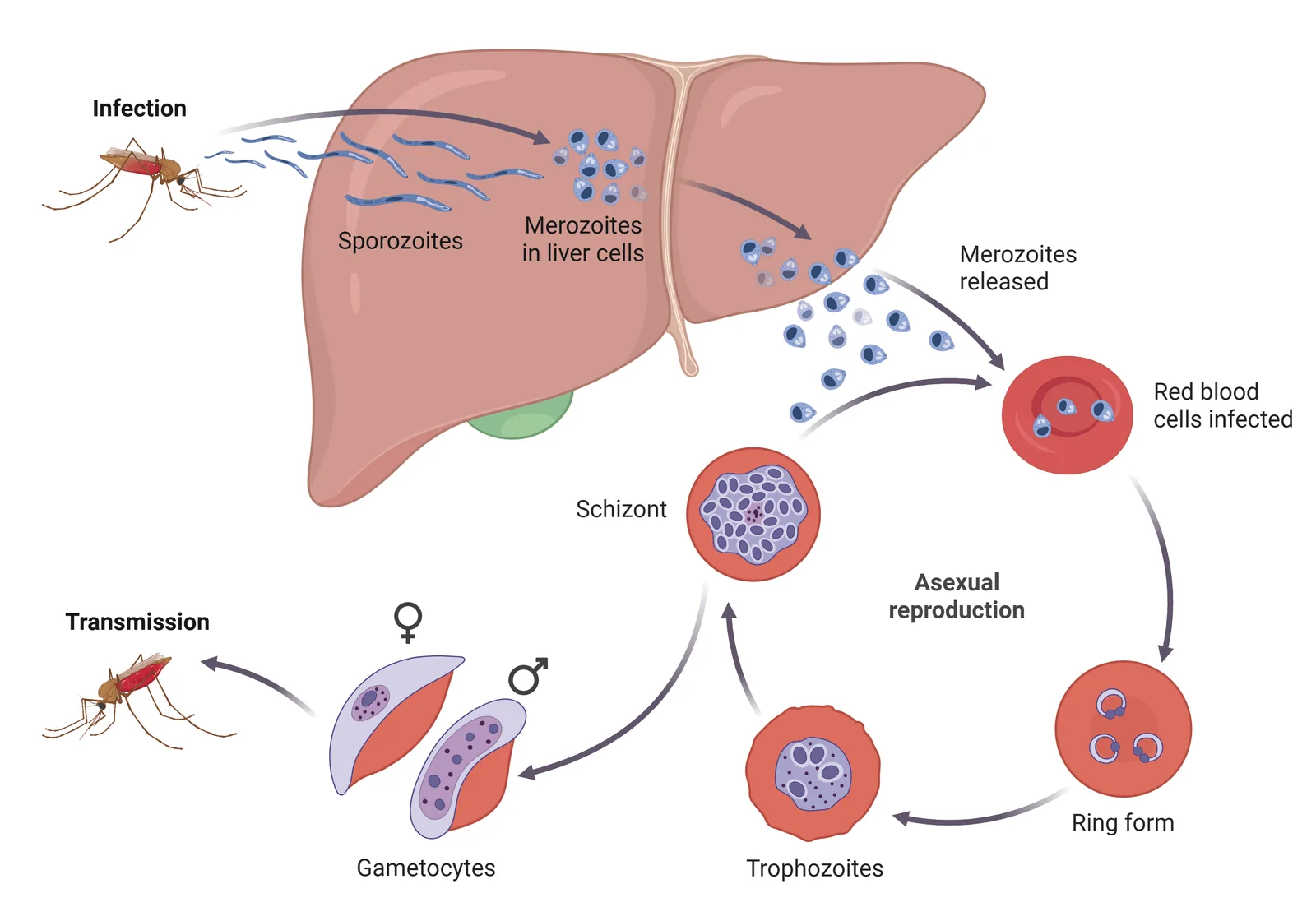
Transmission cycle of malaria Created in BioRender. Image credits: Cerezo S (2026). Artificial intelligence has not been used in image creation.

*P. falciparum* has a complex life cycle (Figure 1) that goes through both sexual and asexual reproduction. Infected *A. gambiae* takes blood meals, which inject sporozoites into the skin. Sporozoites are motile and migrate toward the liver once they enter the bloodstream to escape the immune system of the host [6]. After reaching the liver, sporozoites infect hepatocytes where the first round of asexual reproduction occurs [7]. A multinucleated exo-erythrocytes schizont formed through asexual reproduction ruptures, releasing merozoites into the bloodstream [8]. Once back in the bloodstream, the merozoites invade red blood cells, where a second round of asexual reproduction occurs.

When comparing the different Plasmodium species’ transmission cycles from a clinical point of view, it is important to note key differences between *P. falciparum* and *P. malariae* and *P. vivax* and *P. ovale*. *P. vivax* and *P. ovale* only infect reticulocytes, resulting in a tertian fever cycle. *P. vivax* and *P. ovale* can lay dormant in the liver for months to years in a hypnozoite life form. *P. malariae* exclusively infects mature red blood cells and has a quartan fever pattern. *P. falciparum* infects all forms of red blood cells and thus has an irregular fever cycle. Due to developing chloroquine resistance, *P. vivax*, *P. ovale*, and *P. falciparum* can prove challenging to treat. Thus, developing vector transmission mitigation strategies is paramount to public health safety in endemic regions.

The rate of sexual reproduction is sensitive to environmental factors, and only a small portion of merozoites will commit to the production of sexual progeny and undergo gametocytogenesis [9]. Host-derived lipid lysophosphatidylcholine (LysoPC) has a demonstrated linkage between parasite metabolism and the activation of sexual-stage-specific transcription, followed by gametocyte formation [10]. LysoPC controls *P. falciparum* fate through the repression of parasitic sexual differentiation. Thus, repression of LysoPC promotes sexual differentiation and gametocytogenesis. Additionally, the anti-malarial drug chloroquine has been found to promote gametocytogenesis of *P. falciparum* while inhibiting the asexual growth of the parasite [11].

**Figure 2:**
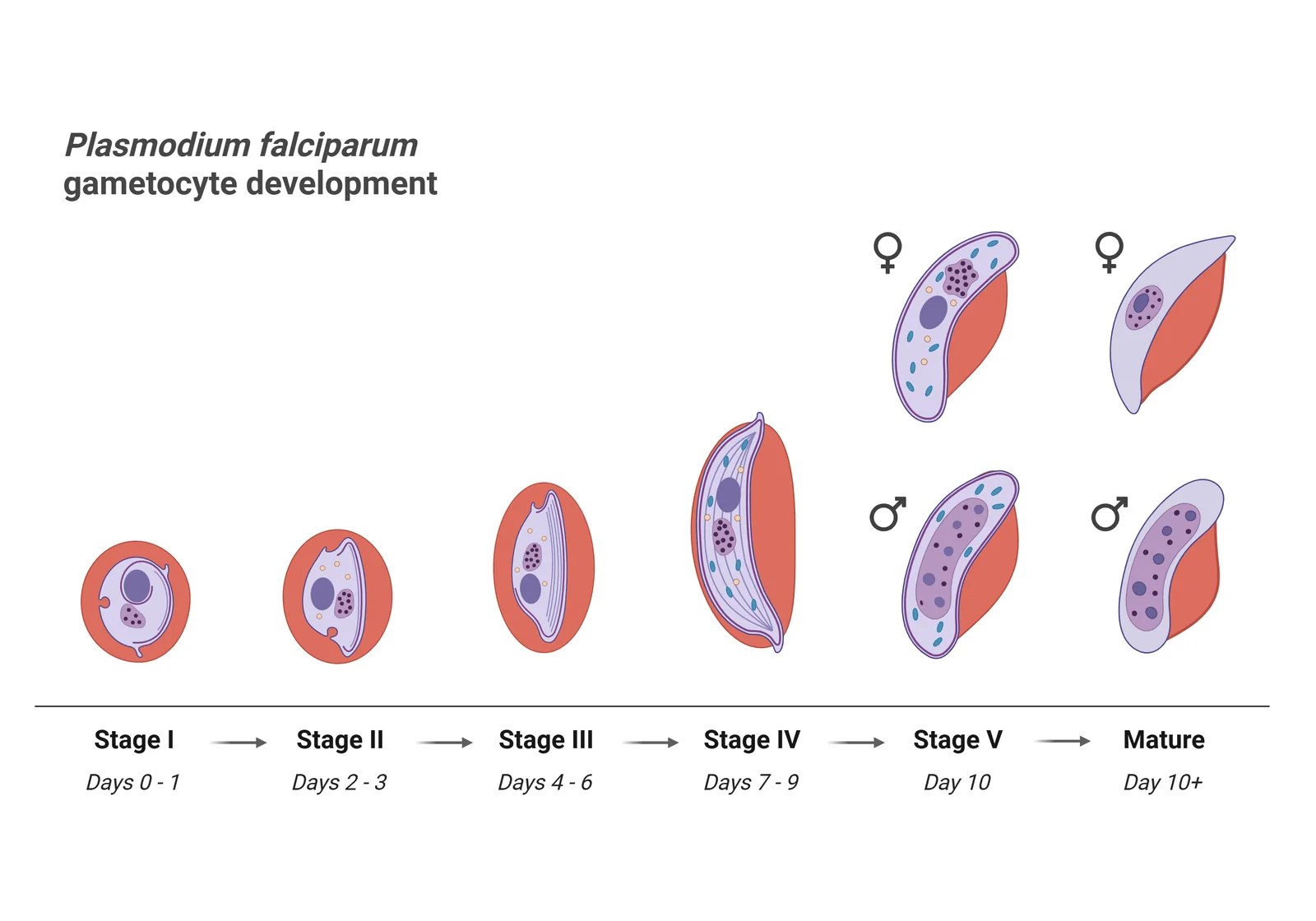
Plasmodium falciparum gametocyte development Created in BioRender. Image credits: Cerezo S (2026). Artificial intelligence has not been used in image creation.

Gametocytes are ingested by *A. gambiae* during the next blood meal. After entering the midgut of the mosquito, proteases are used by gametocytes to exit erythrocytes and begin zygote formation [12]. Male gametocytes differentiate into eight microgametes, while female gametocytes differentiate into one macrogamete. The subsequent fusion of microgamete and macrogamete forms a zygote. Following fertilization, the zygote matures into an ookinete. Two limiting steps in maturation to ookinete from a zygote have been identified: inhibition of the transition from stage I to stage II and arrest in the transition from step III to step IV (Figure 2) [13]. These steps are likely caused by inefficient fertilization and morphological abnormalities, respectively.

Following maturation from a zygote, the ookinete crosses the epithelial layer of the midgut wall, which then further matures to an oocyst. As an oocyst, the parasite undergoes the third round of asexual replication to produce sporozoites. The sporozoites are subsequently released from the oocyst into the hemolymph of *A. gambiae*. Two parasite proteins have been identified to promote the release of sporozoites from the oocyst. Interfering with these oocyst rupture proteins is a potential target for blocking transmission of the parasite [14]. Sporozoites migrate into the hemolymph and to the salivary glands of the mosquito, where they can then be transmitted to the next human host and restart the cycle.

## Review

Approaches to mitigate vector transmission of malaria 

Clustered Regularly Interspaced Short Palindromic Repeats-Based Gene Drives

**Figure 3:**
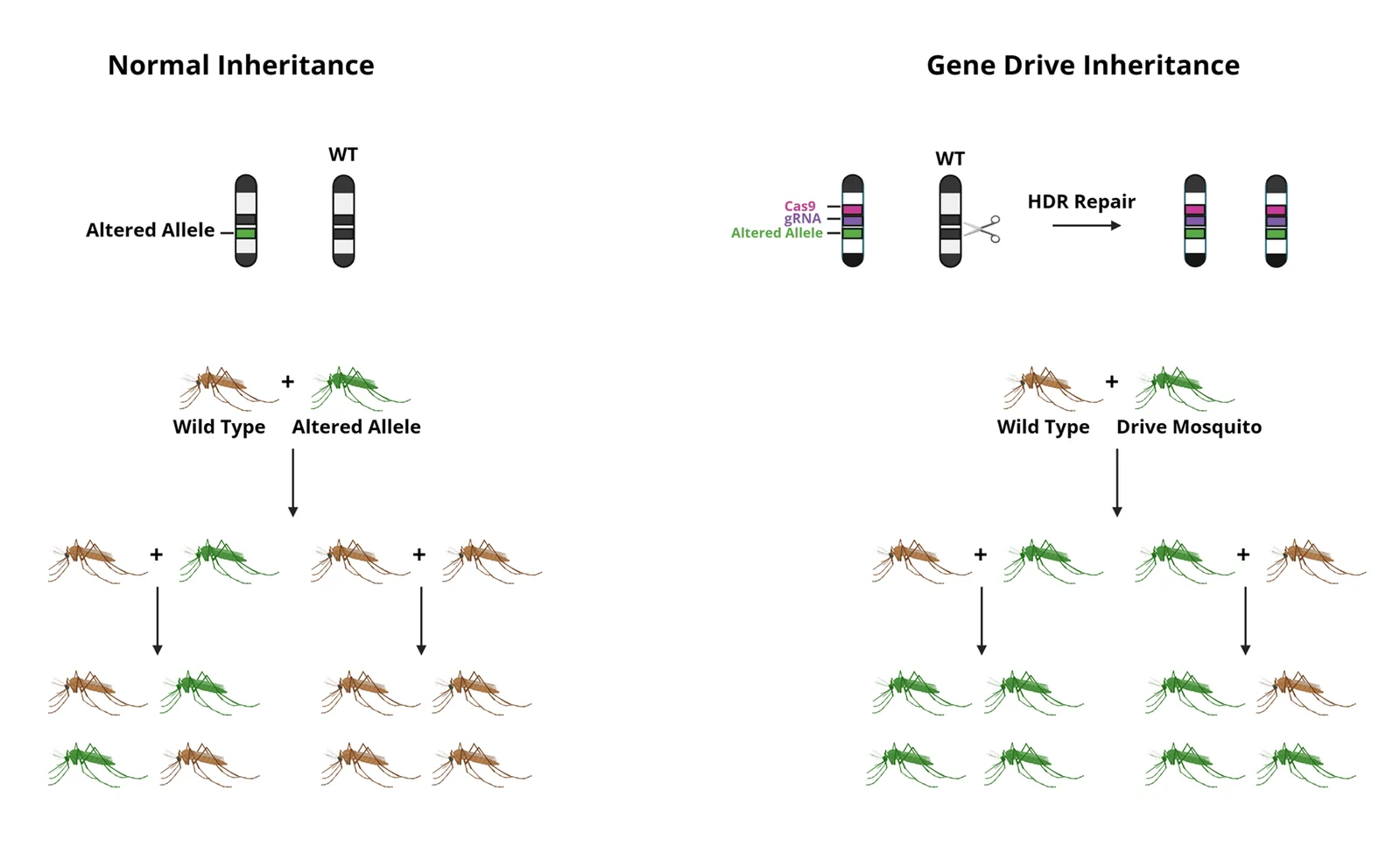
Normal inheritance versus gene drive inheritance Created in BioRender. Image credits: Cerezo S (2026). Artificial intelligence has not been used in image creation.

Clustered regularly interspaced short palindromic repeats (CRISPR)-Cas9 gene drive systems have enabled researchers to harness genetic specificity to introduce transgenes, such as those conferring resistance to Plasmodium or causing sterility, into target organisms. Gene drives promote supramendelian inheritance, allowing these genetic elements to spread rapidly through a vector population. CRISPR-Cas9 gene drive systems rely on CRISPR, which is a bacterial defense mechanism characteristic of bacterial genomes to protect against bacteriophages. These segments of DNA are paired with specific guide proteins known as CRISPR-associated proteins [15]. The Cas9 system specifically uses CRISPR-associated protein 9, an endonuclease enzyme, to induce a double-strand break in the target DNA, allowing genome modifications [16]. Figure 3 illustrates the difference between standard Mendelian inheritance and gene drive inheritance using a driver allele inserted with CRISPR-Cas.

Multiple questions arise when considering which gene drive method to use for the neutralization of *A. gambiae,* such as whether a reduction drive or a replacement drive is going to be used. Some gene drive methods use population suppression in which the vector population is reduced to drive disease transmission down. This typically involves a deleterious allele being spread throughout the population. These alleles can typically affect reproduction of the vector or result in death, but the fitness costs of these methods must be analyzed to ensure proper genetic driving occurs [17]. A replacement drive follows the same base principles as the reduction drive, except the goal is to replace the *A. gambiae* population with a transgenic line that has resistance to Plasmodium.

An advantage of utilizing CRISPR-Cas9 is the specificity of the guide RNA and the targeted DNA sequence. To truly understand the cost-effectiveness of using CRISPR-Cas9 gene drives in the case of *A. gambiae,* we must first understand the *A. gambiae* genome and which genes to target, as efficacy is paramount to a drive’s success. Current trials being conducted with *A. gambiae* have shown success. Target Malaria has created a CRISPR-Cas9 gene drive focusing on impacting mosquitoes’ ability to reproduce. The group specifically focuses on *A. gambiae*, *A. coluzzii*, and *A. arabiensis*. Target Malaria specifically targets the doublesex (dsx) gene and has demonstrated complete population suppression in cage trials [18]. The approach specifically targets a region of the dsx gene that is only expressed in females and is essential for sex determination. This trial demonstrated efficacy in a population suppression drive, which, in turn, has the potential to drive disease transmission down. This study highlights an important concept in selecting a specific gene to target. CRISPR-Cas9 drives are not without fault, and resistant variants have the potential to arise when constructing a drive. By using a gene such as dsx that has functional or structural constraints to *A. gambiae*, we can reduce the development of resistant variants that can affect the driving potential. Two benefits of Target Malaria’s approach using CRISPR-Cas9 gene drives are that this system is self-propagating and that limited releases are required compared to other techniques such as sterile insect techniques (SITs) or repeated insecticide spraying. Moreover, the complete suppression achieved in the cage model suggests the potential for high scalability in low-resource regions. Limitations to CRISPR-Cas9 interventions are contingent upon population migrations. The cage-model approach is limited in the capacity of not accounting for migration that is present in nature. CRISPR-Cas9 systems offer benefits in a cost analysis sense, as new proteins and complicated genes do not need to be developed; instead, the Cas9 system uses a cheaper short RNA guide that can be synthesized through the use of existing technologies [19].

Other examples include X-shedder and homing endonuclease techniques that are low-threshold gene drives. This suggests quick integration of the transgenic line into the population. While this is sure to mitigate costs, there are inherent challenges. The fate of any gene drive implemented into a population is directly linked to the ecology of the surrounding area [20]. The dangers in low-threshold gene drives lie in the fact that if unintended consequences occur, they have the potential to quickly establish within the population and, once established, may be unable to be reversed. Taking steps to mitigate gene-drive spillovers to non-target populations should be carefully considered, keeping in mind negating migration rates as much as possible.

The implementation of a CRISPR-Cas9 gene drive system must demonstrate efficiency to display cost-effectiveness. A CRISPR-Cas9 system alone will not simply eradicate malaria on its own, but it provides a tool that can be used with current control methods that can decrease the cost of disease burden and transmission simultaneously. A mathematical model estimating the most cost-efficient method of using a drive in conjunction with current malaria interventions has demonstrated an estimated cost of gene drive deployment below $7.17 per person per year [21]. This study analyzed the efficiency of using a driving-Y gene drive for malaria elimination in eight provinces in the Democratic Republic of Congo. Gene drive efficacy was critical in the simulation, as at least 95% X-shredder efficiency must be maintained for drive technologies to be viable in a cost-effective manner. While this may seem like an easy answer, there is still the challenge of producing a drive that will work in the field with at least 95% efficacy.

Overall, a CRISPR gene drive system has the potential to be a supplemental tool in the neutralization of malaria as demonstrated by the Congo study [18]. Simply stated, CRISPR alone will not diminish the spread of malaria on its own. A combined approach using current methods such as insecticide-treated nets, indoor residual spraying, and medical interventions in conjunction with a CRISPR-based drive does provide a potential cost-effective method for malaria suppression.

*Maternal-Effect Dominant Embryonic Arrest* *and Underdominance*

Gene drives using genetic technology also have different thresholds. Controllability is a concept critical to the implementation of drive technologies due to public concern about transgenic lines being introduced into the ecosystem. Threshold drives are frequency-dependent gene drives that are considered more controllable [17]. Toxin-antidote-based underdominance and maternal-effect dominant embryonic arrest (MEDEA) synthetic lines use frequency dependence. These drives represent two distinct approaches for altering mosquito populations and have the potential to be kept more local if gene flow is limited between populations. The difference between the techniques is that MEDEA uses a maternally expressed toxin and a zygotically expressed antidote to disrupt embryonic development, thereby allowing for the spread of the drive. In contrast to MEDEA, underdominance relies on heterozygotes having lower fitness than either of the homozygotes, resulting in similar population replacement outcomes as MEDEA.

**Figure 4:**
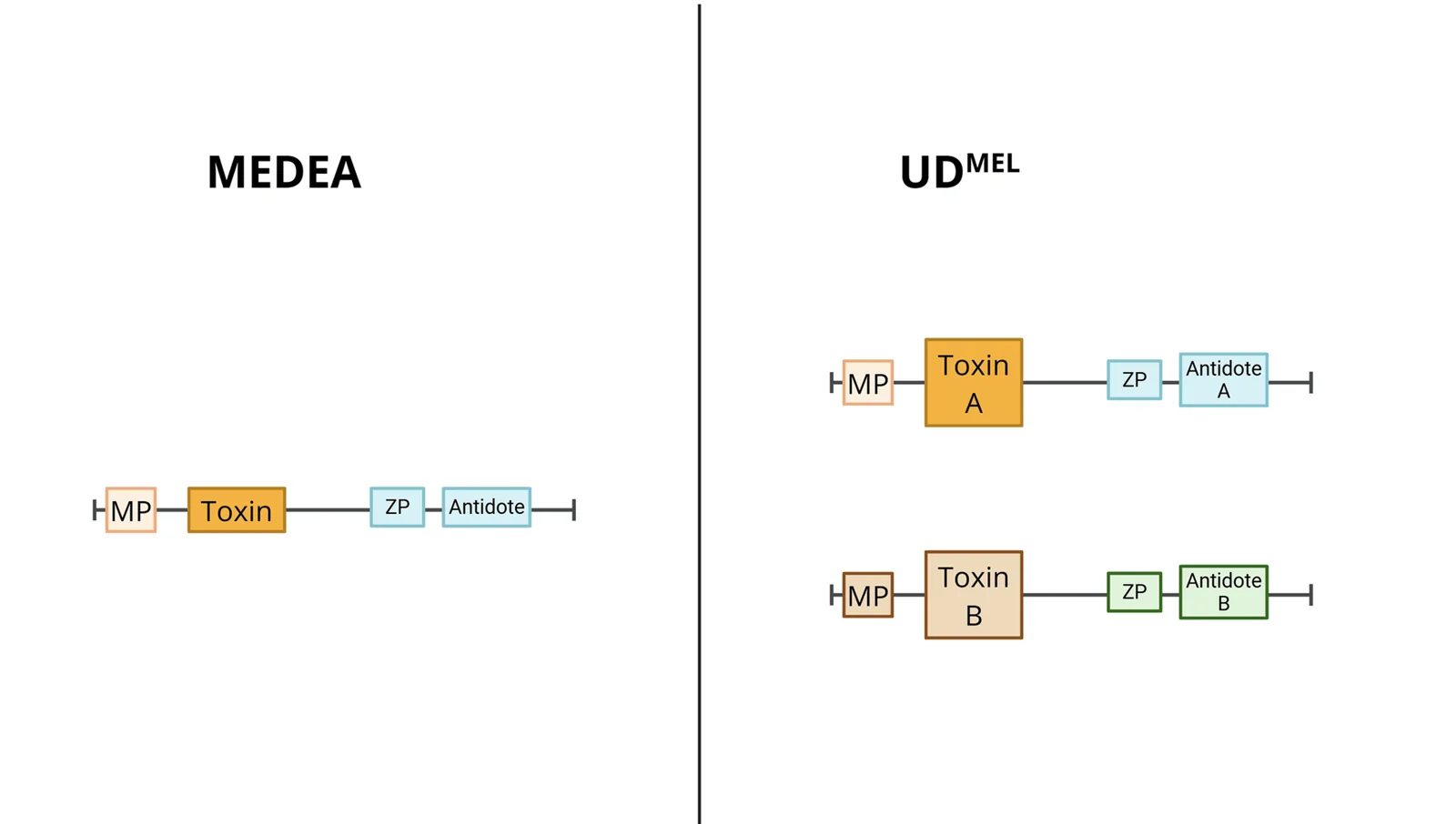
MEDEA and underdominance Created in BioRender. Image credits: Cerezo S (2026). Artificial intelligence has not been used in image creation. MEDEA, maternal-effect dominant embryonic arrest.

MEDEA technology (Figure 4) involves two constructs on the same chromosome. One construct expresses a maternally expressed toxin that disrupts mosquito development, while the other construct expresses a zygotically expressed antidote that counteracts that toxin’s effect. Therefore, individual mosquitoes in the population that do not inherit the maternally expressed toxin and the antidote together will be eliminated [22], thereby creating a selective advantage for the mosquitoes carrying the drive.

Underdominance technology (Figure 4) involves two constructs on different chromosomes in which each carry a maternally expressed toxin and a zygotically expressed antidote that can suppress the toxin from the other chromosome. Since the fitness of heterozygotes is lower than that of homozygotes, the outcome of population replacement is similar to that of MEDEA technology.

The two systems offer the ability to be more controllable when compared to other gene drive mechanisms, but a drawback with using these systems is the need to release large numbers of mosquitoes into an area, which may not be a cost-effective method. Both methods rely on thresholds of mosquitoes being maintained for driving to occur. The threshold could act as a potential stop mechanism for the drive. Another stop mechanism for the drive could be to release more wildtype mosquitoes in target areas. This can negatively impact individuals in endemic regions, as it will likely increase disease transmission and disease burden. Efficiency and controllability are two massive factors when designing drive technologies and, consequently, may be inversely related [20].

It should also be considered that when using drives such as MEDEA and underdominance, there is the potential for altering behavior among target species such as with *A. gambiae* mosquitoes. Individuals living in target regions and stakeholders should be aware of the potential ecological implications of using these drive technologies. Due to the vast span of *A. gambiae* distribution, using threshold drives may prove to be difficult and expensive. Progress has been made in the development of MEDEA and underdominance technologies for *A. gambiae,* but there is still a lot that needs to be researched regarding ecological implications.

Transposable Elements

Transposable elements or transposons are another genetic avenue available for the modification of the *A. gambiae* genome. Transposons (TEs) are mobile genetic elements found in organisms that make up a portion of the genome. Due to their selfish nature and ability to integrate into the host organism's genome via transposition, transposons offer a method for the introduction of potential anti-pathogen genes or the ability to create a gene drive.

Transposable elements fall into different classes. Class I transposons are believed to have originated from ancient retroviruses and replicate via an RNA intermediate that is reverse-transcribed back into cDNA and then integrated into the organism's genome [23]. Class II transposons differ in that they use a copy-and-paste mechanism where they excise themselves from one location and move to a new location within the genome [16]. Both classes of transposable elements are highly mutagenic.

**Figure 5:**
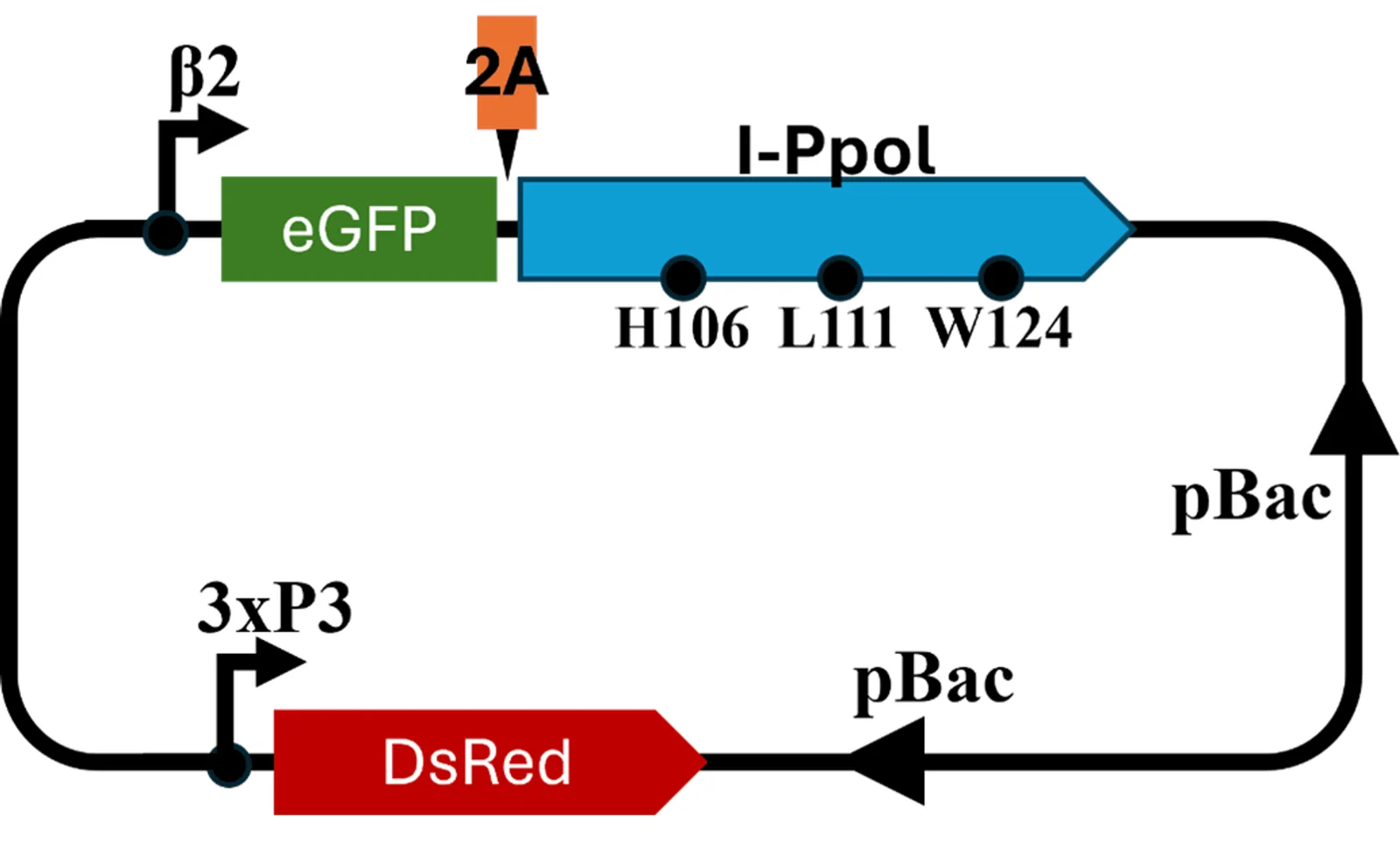
Galizi piggyBac I-Ppol construct [26]

Current efforts with mosquitoes use class II transposable elements, which use inverted terminal repeats with a transposase-encoding open reading frame. In the case of *A. gambiae,* the transposon Herves has been thought to be a potentially successful transposon able to integrate into the genome [24]. The transposable element piggyBac (Figure 5) has proven to be a viable option for *A. gambiae* transformation. To use these transposons to insert genes into *A. gambiae*, genetic segments of interest are placed between inverted repeats to be moved via the transposons from a plasmid vector into a mosquito genome. Transposable elements such as piggyBac offer utility in the ease of manipulation and their ability to allow users to investigate multiple locations within the genome for potential target sites [25]. This contrasts with the CRISPR system’s site specificity.

The use of the piggyBac transposable element has been shown to demonstrate effectiveness in controlling *A. gambiae*, specifically in cage trials. A synthetic sex distortion system was constructed using the piggyBac transposon, resulting in caged mosquito populations with an above 95% male distribution [26]. The piggyBac sex distortion system cleaved the paternal X chromosome, thus leaving only male progeny. This effectively decreased the cage population due to a lack of female mosquitoes. In terms of its efficacy, this method is similar to the CRISPR-Cas9 X-Shredder examined in the Congo [21].

This calls into question the cost-effectiveness of producing a TE gene drive system compared with a CRISPR-Cas9 system, as both demonstrated the same efficacy within cage trials. If the costs of the CRISPR system and the transposon system are relatively the same, then the ecological and social aspects of the two technologies should be weighed. The public may be reluctant to use transposons due to their mutagenic abilities and ability to jump virtually anywhere in the genome, while CRISPR systems are targeting specific locations within the genome.

Wolbachia

**Figure 6:**
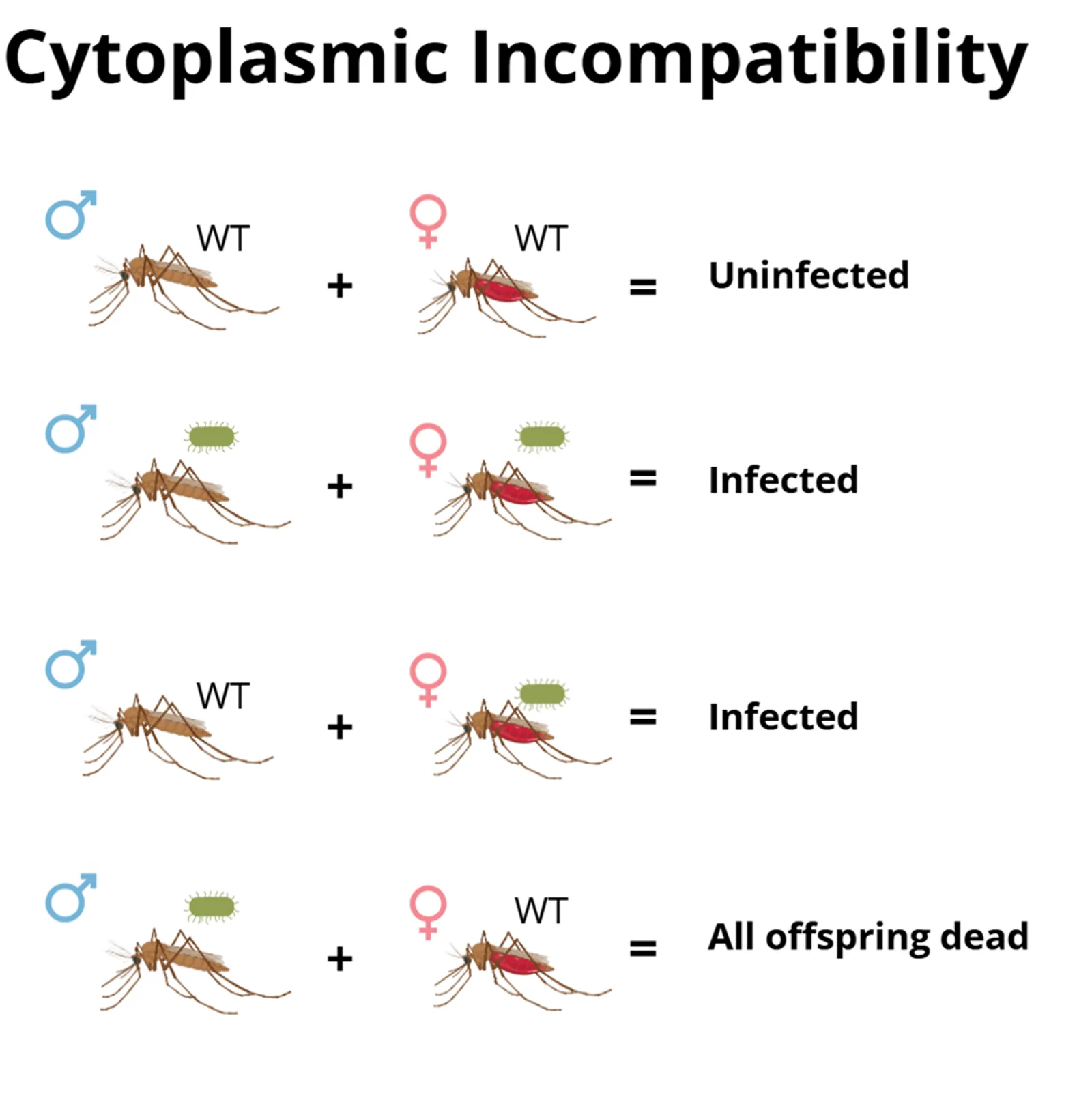
Cytoplasmic incompatibility depicting offspring survival and infection results dependent on sex infected Created in BioRender. Image credits: Cerezo S (2026). Artificial intelligence has not been used in image creation.

Wolbachia is an endosymbiont bacterium that is found naturally in a large number of insect species and is maternally inherited [27]. Infection by Wolbachia causes a wide range of abnormal reproductive phenotypes, with the most well-known and applicable being cytoplasmic incompatibility (Figure 6). Cytoplasmic incompatibility rendered by Wolbachia is a phenomenon where infected males are sterile when mating with uninfected females but not with infected females [28].

Cytoplasmic incompatibility strives to systematically reduce the population size of mosquitoes. Inducing Wolbachia infections to reduce vector populations has been successfully implemented in *Aedes aegypti* mosquitoes to reduce transmission of dengue, chikungunya, Zika, and yellow fever. Up to a 95.55% reduction in female *A. aegypti* populations has been estimated to occur in large-scale suppression of Wolbachia-infected male release trials [29]. One of the most important parameters to consider when conducting large-scale Wolbachia-infected male releases is ensuring that female contamination does not occur. The viability of offspring becomes restored if the female is also infected with Wolbachia [30].

The primary method for inducing Wolbachia infection into vector lines is through microinjection of embryos or adults. This technique, known as transinfection, is the mechanical transfer of symbionts into their host. Microinjection of embryos is used far more often than that of adults. Pre-blastoderm embryos are injected with Wolbachia with a fine needle and micromanipulator [31]. Injection into pupae is also possible and has been used in certain wasp species [30]. However, for successful vector suppression, Wolbachia must cross to the germline, and it is more easily achieved through embryonic microinjection.

A study based at Michigan State University analyzed whether Wolbachia-infected mosquitoes had protective mechanisms against infection from human pathogens and discovered that Wolbachia blocks *P. falciparum* in mosquitoes that do not naturally carry it, such as *A. stephensi* [32]. The mechanism of protection is not well understood; however, it is speculated that Wolbachia makes changes to mosquito immunity, metabolism, and host cellular mechanisms responsible for the production of proteins, which are used and exploited by pathogens during infection.

Creating a large-scale intervention program around the incompatible insect technique is no easy task. The study that accomplished a 95.55% reduction in population released 14.4 million males over an area spanning 293 hectares, which only encompasses a little over 1 square mile [29]. The study was carried out in a densely populated city (Fresno, California), so it is important to consider subsequent increases in vector density that are likely present.

The cost of control for *Culex quinquefasciatus* in Hawai’i at the Alak’i Wilderness Reserve is estimated at roughly $1.75 million for the first year and around $750,000 subsequently [33]. *Culex quinquefasciatus* is an ecological hazard in Hawai’i. *Culex quinquefasciatus* vectors *P. relictum,* which causes avian malaria [34]. Important differences that could cause disparities in cost compared to the Fresno study are that the Alak’i Wilderness Reserve is at a heightened elevation, has much lower human interactions, contains different area sizes, and has different release levels of incompatible males. In Hawai’i, approximately one million incompatible males are released weekly, while Fresno released 14.4 million for the entire year. Based on these differences, one could infer a lower cost of the program in Fresno than in Hawai’i.

While both interventions do provide a pragmatic example for introducing the incompatible insect technique into populations, the ever-difficult challenge of distribution and high production costs cannot be understated. *A. gambiae* mosquitoes inhabit an area estimated at 16.58 million square kilometers [35]. Under the current methods, rapid introduction to combat malaria infections is just not feasible. The World Mosquito Program calls for leaning on engagement strategies with local communities to introduce Wolbachia-infected mosquitoes into the population through scheduled adult and egg releases, the development of transport apps, drone utilization to diminish operating costs, and increase population reduction over a quicker period of time [36].

Paratransgenesis

Another vector control strategy similar to Wolbachia is paratransgenesis. Paratransgenesis involves genetically modifying symbionts of insect vectors to reduce their ability to transmit pathogens. Paratransgenesis was initially investigated in the gut of the *Rhodnius prolixus*, a kissing bug that belongs to the Reduviidae family and transmits Chagas disease [37]. The initial strategy investigated the production of the antimicrobial peptide cecropin A by the bacterium *Rhodococcus rhodnii* to suppress vectoring of *Trypanosoma cruzi* [38].

Early efforts to develop a paratransgenesis approach in controlling vectoring of malaria were built upon the hypothesis that *Pantoea agglomerans*, a bacterial symbiont of Anopheles, could be engineered to secrete anti-Plasmodium effector proteins. Plasmids that include pectate lyase from *Erwinia carotovora* and hemolysin A from *Escherchia coli* were introduced into *P. agglomerans* and *E. coli* [39]. Following attempts at a paratransgenesis approach were performed through genetic engineering of *E. coli* to display two anti-Plasmodium effector molecules, salivary gland and midgut peptide 1, and phospholipase-A2 on its outer membrane [40]. *E. coli* does not survive well in the mosquito midgut, thus hindering the efficacy of this approach. Recently, efforts have shifted toward *P. agglomerans*. *P. agglomerans* was genetically engineered to secrete anti-Plasmodium effector molecules that inhibit parasite development. The *E. coli* hemolysin A secretion system was used to secrete scorpine, a potent antiplasmodial peptide, and enolase-plasminogen interaction peptide, which prevents plasminogen binding to the ookinete surface [41]. These engineered *P. agglomerans* strains inhibited the development of *P. falciparum* by up to 98%.

In the development of paratransgenic approaches, it is important to identify a symbiotic bacterium with minimal fitness cost to the mosquito for successful implementation. The team that genetically modified *P. agglomerans* discovered a previously unknown bacterial strain, Serratia AS1, which shows 99% similarity to *Serratia marcescens* [42]. AS1 infection displayed little to no effect on life span and was non-influential in blood-feeding behavior in *A. gambiae* and *A. stephensi* [43]. Additionally, the Serratia HasA exporting system was used to test anti-Plasmodium effectors. Strains that expressed scorpine and multiple effector proteins reduced oocyst load by up to 93%. The advantage of paratransgenesis over genetically transformed mosquitoes is that the transformed symbionts have the ability to colonize a range of different insect vector species, suggesting low maintenance costs following introduction. In addition, this approach can be combined with gene drives for self-sustaining spread and can reduce malaria transmission without affecting mosquito population size; therefore, it may be more likely to be accepted by the public.

SIT

SIT constitutes the mass release of male mosquitoes that have been sterilized through radiological or chemical means to mate with wild females, thereby resulting in no offspring [44]. The most common form of sterilization in SIT programs has been exposing germ cells to gamma radiation, thereby inducing chromosomal breaks that cause a breakdown in the genome following fertilization and death in early embryogenesis [36]. Generally, *A. gambiae* females mate just once [45]. This allows the mass-released sterile males to outcompete non-sterile wild-type males [46]. The success of these implementations is contingent upon wild-type females and mass-released males readily mating and depends on a variety of factors. Challenges with SIT mostly come from the fact that repeated mass releases are necessary due to success being threshold-dependent; however, the benefit in this challenge is the localized and controlled nature of it.

Recent progress has been made in developing transgenic interventions for *A. gambiae* for SIT. The challenge in developing an effective sterilization technique is to ensure sufficient germ cell damage to cause sterility yet minimize the somatic damage so that overall fitness is not minimally affected, ensuring continuing mating competitiveness for transgenic mosquitoes. Other applications for sterility include using homing endonucleases to selectively destroy the X chromosome, preventing female progeny, and using RNA interference to lower male sperm production [47]. A successful application of X-shedder is the insertion of DNA encoding I-PpoI, a homing endonuclease gene, from the slime mold *Physarum polycephalum* that specifically cleaves the ribosomal DNA repeats present on the X chromosome, thereby creating sterile males [48]. For any SIT approach, population suppression is highly dependent upon the number of released males. After a certain proportion is released, additional males have diminishing returns on population suppression due to lethal allele saturation [20]. Determining the optimal proportion necessary for SIT is important for the effective utilization of funding for large-scale future projects.

Community Engagement and Regulation

For any vector control program, it is important to understand the values, customs, and norms of the population that will be directly affected by the intervention. Oftentimes, interventionists come from significantly different world viewpoints than those that will be affected. Developing relationships with locals to gain an understanding of what will be culturally accepted is an important part in gaining trust with communities. Additionally, engagement with local individuals aimed at providing education on disease transmission mitigation can give locals a sense of control, making participation in the implemented program(s) more likely if necessary. Both thought chains are important in determining what is a good theoretical implementation and what is a good pragmatic implementation.

*A. gambiae* presides over a wide range of Sub-Saharan Africa. In such a culturally diverse region, it would be nearly impossible to discover and implement a successful suppression strategy for malaria. Genetic engineering carries certain stigmatizations, and creating sterility within a population carries others. The especially high genetic diversity of *A. gambiae* calls for especially cautious genetic analysis when using gene drives to ensure success is not jeopardized by polymorphisms [49]. Mitigating the risk of creating ecological hazards or the accidental increase in vector competence should be at the forefront of any implementation program.

There is a fine balance that must be considered between the pressures of stakeholders and the ethical considerations to be made in effectively serving an at-risk population. In practice, three considerations for operationalizing stakeholder engagement have been used for pragmatism. Adapting to stakeholder preferences, inclusiveness, empowerment, and accountability have been effective steps to take in successful community engagement, as shown by the Target Malaria project in Burkina Faso, Mali, and Uganda. To achieve effective community engagement, it is important that the strategy seeks to enable the mobilization of the local population, as any effective malaria control program will require community participation [50].

Researchers walk a delicate line when designing and implementing control strategies for malaria. Adhering to stakeholder guidelines while delivering ethically sound research in the field is a challenge that requires open dialogue on many ends. The best engagement protocols occur early and often and promote a cooperative environment on all ends through the three groups of interest: stakeholders, the research team, and the local community [51].

In 2017, the World Health Assembly developed the “Global Vector Control Response (GVCR) 2017-2030” seeking to promote an integrated approach to the control of vector-borne diseases [52]. Programs under the GVCR should lead vector control needs assessment to appraise current capacity, define the requisite capacity to conduct the proposed intervention, identify opportunities for improved efficiency in vector control delivery, and guide resource mobilization.

Recently, several recommendations through online workshops have been made to advance environmental risk assessments for gene drives [53]. Common themes in the recommendations made show a desire for an expanded dialogue to include more community engagement and to bring in experts in different domains to consider more angles. Additionally, the recommendations call for a more expanded protocol in the assessment of risks to prevent adverse consequences.

Due to most *A. gambiae* intervention programs being done internationally, there is not a simple national governing regulation to have oversight into all aspects of a project. While there can be oversight on the research carried out at the place of research by national regulatory agencies, the location of the programs being implemented is often done in countries without such governmental infrastructure. Additional ethical burden and responsibility are thus placed on research teams to act in a humanistic manner. Domestically, in the United States, the Environmental Protection Agency (EPA) and the Food and Drug Administration (FDA) would have oversight of different vector control programs. The FDA reviews products intended to prevent mosquito-borne disease in humans or animals, and the EPA reviews products intended to reduce the population of mosquitoes.

Discussion

Malaria is a complex disease cycle affecting 249 million people in 85 countries in 2023, according to the World Health Organization [54]. This paper has demonstrated that there are many intervention techniques being researched to mitigate disease transmission by vectors, but each one possesses its own unique challenges (Table 1). Those challenges include the cost of the intervention, the inability to control migration, unique ecological challenges specific to a location, or simply a lack of public approval. There is not a single stop-all solution. 

**Table 1:** Comparative analysis of transgenic and genetic-based approaches to reduce Anopheles gambiae malaria transmission CRISPR, clustered regularly interspaced short palindromic repeats; MEDEA, maternal-effect dominant embryonic arrest.

Intervention	Mechanism of action	Primary objective	Advantages	Limitations
CRISPR-based gene drives	Uses CRISPR-Cas9 to bias inheritance of engineered genes, allowing rapid spread through mosquito populations. Gene drives may suppress mosquito populations or replace them with malaria-refractory mosquitoes.	Population suppression or population replacement	Highly efficient inheritance; self-sustaining; potential for long-term control with relatively few releases; species-specific targeting	Evolution of resistant alleles; ecological uncertainty; ethical and regulatory concerns; potential transboundary spread
MEDEA and underdominance systems	Synthetic gene drives systems that promote inheritance through maternal-effect lethality (MEDEA) or heterozygote disadvantage (underdominance), enabling controlled spread of engineered traits.	Population replacement	More geographically confined than CRISPR gene drives; potentially reversible; improved containment	Less efficient spread than CRISPR gene drives; requires high release thresholds; technically complex
Transposable elements	Mobile genetic elements insert desired genes into mosquito genomes to express antiparasitic effector molecules or other beneficial traits.	Population replacement	Stable transgene integration; valuable research platform for developing genetically modified mosquitoes	Random genomic insertion may affect gene expression; limited efficiency; generally requires additional drive systems for population-wide dissemination
Wolbachia	Introduction of Wolbachia endosymbiotic bacteria induces cytoplasmic incompatibility and may reduce Plasmodium development or interfere with mosquito reproduction.	Population replacement and transmission reduction	Self-propagating after establishment; reduces reliance on insecticides; environmentally sustainable	Stable colonization of *Anopheles gambiae* remains challenging; effectiveness may vary by strain and environment.
Paratransgenesis	Genetically engineered symbiotic microorganisms colonize mosquitoes and produce molecules that inhibit Plasmodium development within the vector.	Transmission blocking	Does not require permanent modification of the mosquito genome; flexible platform; species-specific	Difficulty maintaining engineered microbes in wild populations; ecological and regulatory uncertainties
Sterile insect technique	Large numbers of sterile male mosquitoes are released to mate with wild females, producing no viable offspring and reducing mosquito population size. Sterility may be induced through irradiation or genetic engineering.	Population suppression	Species-specific; environmentally friendly; no persistence of modified genes in the environment; well-established vector control strategy	Requires continuous mass rearing and repeated releases; high operational costs; less effective over large geographic areas

This paper focused mostly on ways to combat malaria by altering the vector population or genome. Other approaches to consider are interventions that target *P. falciparum* at different life stages. Research has already shown that drugs targeting both histone and non-histone epigenetic modifiers are successful in both the asexual and sexual development of *P. falciparum*.

When imposing a vector control measure, it is important to take into account the sustainability, feasibility, and cost of the methodology. Our review of the literature suggests that the most cost-efficient approach to vector control of malaria lies in an approach that combines a CRISPR-Cas9 gene drive targeting dsx with paratransgenesis using *P. agglomerans* to secrete anti-Plasmodium effectors, specifically scorpine. This approach offers scalability from CRISPR-Cas9 following initial introduction and protection of the transgenic population from *P. agglomerans,* based upon the lack of fitness cost from paratransgenesis. This approach figures to be cost-efficient with the ability to offer long-term malaria control in endemic areas, with minimal recurrent costs and maintenance to the system that are observed with SIT and incompatible insect technique. Initial costs are likely to be less than what is observed with underdominance and MEDEA techniques, which require large amounts of mosquitoes to be released initially to achieve threshold, in addition to their maintenance costs to maintain threshold. Finally, the CRISPR-Cas9 approach yields favorability to approaches using transposable elements due to the controlled production of progeny. 

At the forefront of the public health agenda should be continued and expanded outreach to at-risk communities to provide anti-malarial drugs such as chloroquine, as well as bed nets. Educating populations to eliminate standing water after rainfall, which will remove a breeding ground for *A. gambiae,* should also be done. In terms of engagement, these interventions are already well-received by communities. Continuing these approaches can further build trust with these communities to open the doors for successful implementation of the methods we have discussed. These approaches are currently more pragmatic than any of the vector suppression techniques we have discussed, along with being far more affordable. While chloroquine can be expensive, artemisinin-based combination therapies have been proven effective at a fraction of the cost, at roughly $3 per treatment. Additionally, bed nets run around $5 per unit. While it cannot be understated that three billion individuals are at risk of malaria compared with the roughly million dollars annually it takes to run an incompatible insect technique intervention plan for a square mile, the per capita currently favors increasing availability of bed net and anti-malarial drug supplies. 

## Conclusions

The emphasis on practicality does not diminish the value of ongoing scientific innovation in vector control. At present, the most practical allocation of funds in a crisis is through distribution of the supplies previously discussed, due to the high cost and uncertainty of variables from gene drive and transgenic measures. These methods are moderately efficacious, cheap, and require a low level of literacy to employ properly. 

While emerging methods offer significant long-term potential, they currently carry higher costs and uncertainties. Ultimately, no single strategy is sufficient. The most effective approach to reducing the malaria burden likely lies in combining immediate, community-based interventions with continued research and the integration of advanced technologies.
